# Mr. Evelyn Pocklington

**Published:** 1911-08-12

**Authors:** 


### OBITUARY.

The death is announced of Mr. Evelyn Pocklington, M.R.C.S., late of Wimbledon. He studied at St. Bartholomew's Hospital, took his M.R.C.S. in 1860, and his L.S.A. in 1861. He was surgeon to the Wimbledon Cottage Hospital, and to the National Rifle Association.

